# AVEN: a novel oncogenic biomarker with prognostic significance and implications of AVEN-associated immunophenotypes in lung adenocarcinoma

**DOI:** 10.3389/fmolb.2023.1265359

**Published:** 2023-10-16

**Authors:** Dengxia Fan, Moses Yang, Hye Jung Lee, Jeong Hee Lee, Hong Sook Kim

**Affiliations:** Department of Biological Sciences, Sungkyunkwan University, Suwon, Republic of Korea

**Keywords:** AVEN, lung adenocarcinoma, immune infiltration, prognostic biomarker, prognostic model

## Abstract

**Introduction:** AVEN, an apoptosis and caspase activation inhibitor, has been associated with adverse clinical outcomes and poor prognosis in Acute myeloid leukemia (AML). Targeting AVEN in AML improves apoptosis sensitivity and chemotherapy efficacy, making it a promising therapeutic target. However, AVEN’s role has not been studied in solid tumors. Therefore, our study investigated AVEN as a prognostic biomarker in a more comprehensive manner and developed an AVEN-derived prognostic model in Lung adenocarcinoma (LUAD).

**Method:** Pan-cancer analysis was performed to examine AVEN expression in 33 cancer types obtained from the TCGA database. GEPIA analysis was used to determine the predictive value of AVEN in each cancer type with cancer-specific AVEN expression. Lung Adenocarcinomas (LUAD) patients were grouped into AVEN^high^ and AVEN^low^ based on AVEN expression level. Differentially expressed genes (DEGs) and pathway enrichment analysis were performed to gain insight into the biological function of AVEN in LUAD. In addition, several deconvolution tools, including Timer, CIBERSORT, EPIC, xCell, Quanti-seq and MCP-counter were used to explore immune infiltration. AVEN-relevant prognostic genes were identified by Random Survival Forest analysis via univariate Cox regression. The AVEN-derived genomic model was established using a multivariate-Cox regression model and GEO datasets (GSE31210, GSE50081) were used to validate its prognostic effect.

**Results:** AVEN expression was increased in several cancer types compared to normal tissue, but its impact on survival was only significant in LUAD in the TCGA cohort. High AVEN expression was significantly correlated with tumor progression and shorter life span in LUAD patients. Pathway analysis was performed with 838 genes associated with AVEN expression and several oncogenic pathways were altered such as the Cell cycle, VEGFA-VEGFR2 pathway, and epithelial-mesenchymal-transition pathway. Immune infiltration was also analyzed, and less infiltrated B cells was observed in AVEN^high^ patients. Furthermore, an AVEN-derived genomic model was established, demonstrating a reliable and improved prognostic value in TCGA and GEO databases.

**Conclusion:** This study provided evidence that AVEN is accumulated in LUAD compared to adjacent tissue and is associated with poor survival, high tumor progression, and immune infiltration alteration. Moreover, the study introduced the AVEN-derived prognostic model as a promising prognosis tool for LUAD.

## Introduction

LUAD (Lung Adenocarcinoma) is a subtype of non-small cell lung cancer (NSCLC) that arises from the glandular tissue of the lungs. It is the most common type of lung cancer, accounting for approximately 40% of all cases of NSCLC (Gelatti et al., 2019). In recent years, there have been significant advances in diagnosing and treating LUAD. For example, targeted therapies have been developed to specifically target the genetic alterations that drive the growth of cancer cells in individual patients (Chan and Hughes, 2015; Skoulidis and Heymach, 2019). Immunotherapy has also emerged as a promising treatment option for LUAD. Despite the advances in diagnostic and therapeutic methods implicated in clinical studies, these treatments have been shown to benefit a limited pool of patients. Thus, it is essential and urgent to find the potential and valuable biomarkers for diagnosis, prognosis, and targets for therapy in cancers.

AVEN (Apoptosis, caspase activation inhibitor) is a protein that plays a crucial role in inhibiting apoptosis and promoting cell survival. It binds to anti-apoptotic Bcl-2 family member, B-cell lymphoma-extra-large (Bcl-xL) specifically that retain anti-apoptotic activity. It also interacts with caspase regulator, apoptotic protease activating factor 1 (Apaf-1) (Chau et al., 2000) and prevents Apaf-1 mediated caspases activation (Chau et al., 2000). *In vivo* experiments showed that AVEN knockdown reduced tumor growth and in turn increased apoptosis of hematopoietic neoplasms (Eißmann et al., 2013). In clinical studies, it is reported that AVEN is overexpressed in acute lymphoblastic leukemias/lymphoma patients and associated with poor prognosis (Melzer et al., 2012; Eißmann et al., 2013). Indeed, AVEN expression is significantly higher in recurrent patients (Choi et al., 2006). With previous findings being limited to cancer of blood and bone, the correlation of AVEN expression with prognosis and immune infiltration in different cancers remain unclear.

We first screened the oncogenic role of AVEN in pan-cancer and found that AVEN is highly expressed in Colon adenoma (COAD), Kidney renal clear cell carcinoma (KIRC), Kidney renal papillary cell carcinoma (KIRP), Lung adenocarcinoma (LUAD), Lung squamous cell carcinoma (LUSC), and Thyroid carcinoma (THCA) compared to normal tissue. Interestingly, AVEN overexpression was associated with poor survival only in LUAD patients. Consistently, high tumor progression and reduced levels of B cell infiltration was observed in AVEN overexpressing LUAD patients. AVEN-associated genes and pathways were studied to gain valuable insight of the characteristics and function of AVEN. Furthermore, we developed an AVEN-derived prognostic model in an attempt to provide a promising prognosis tool for LUAD.

## Materials and methods

### Pan-cancer analysis and TCGA data processing

The AVEN mRNA expression in pan-cancer was analyzed by the GSCA web tool (http://bioinfo.life.hust.edu.cn/GSCA). GEPIA (Li et al., 2021) (http://gepia.cancer-pku.cn/) web tool was applied for the survival analysis in COAD, KIRC, KIRP, LUAD, LUSC and THCA. In order to conduct a more detailed investigation into the role of AVEN in LUAD, RNA-seq data and clinical data in LUAD patients derived from TCGA database were obtained from the UCSC Xena website (http://xena.ucsc.edu/). Log2(FPKM+1) value obtained from RNA-seq data was converted to TPM (Transcripts Per Million) value. Subsequently, patients with the highest 25% of AVEN expression were categorized into AVEN^high^ group, and patients with the lowest 25% of AVEN expression were classified into the AVEN^low^ groups. Overall survival analysis was performed in AVEN^high^ and AVEN^low^ groups.

### LUAD patient characteristic analysis

To compare the characteristics between AVEN^high^ and AVEN^low^ patients, clinical data was downloaded from the UCSC Xena website, including age, TNM classification, gender, radiation therapy status, race, AVEN expression, and smoking status. The R package moonBook was exploited to visualize characteristics of patients between AVEN^high^ and AVEN^low^ groups.

### Driver genes alteration analysis

The whole exome data of LUAD patients was downloaded from the cBioportal (Cerami et al., 2012; Gao et al., 2013) to examine genetic alterations (https://www.cbioportal.org/). Driver genes such as TP53, EGFR, KRAS, ERBB2, BRAF, ALK, RET, FGFR3, NTRK3 and ROS1 were selected, and their alteration pattern was examined in AVEN^high^ and AVEN^low^ LUAD patients. The R package ComplexHeatmap was used to generate an oncoprint plot.

### Identification of DEGs and functional enrichment analysis

Spearman’s rank correlation test, which works with rank-order variables instead of raw data value of the variables, was used to obtain differentially expressed genes (DEGs) between AVEN^high^ and AVEN^low^ group. Genes with an absolute R-value>0.4 were used for pathway analysis by ConsensusPathDB (Kamburov et al., 2009) (http://cpdb.molgen.mpg.de/MCPDB). Significantly altered pathways were selected with the criteria of *p* < 0.05 and were visualized using SRplot (https://www.bioinformatics.com.cn/srplot). Genes in Cell cycle and VEGFA-VEGFR2 pathways were further visualized by using the R package ComplexHeatmap. GSEA analysis was performed to compare the pathway enrichment between AVEN^high^ and AVEN^low^ groups by using these gene sets as references: “SHEDDEN_LUNG_CANCER_POOR_SURVIVAL_A6″, “HallMARK_MTORC1_SIGNALINF”, “HALLMARK_EPITHELIAL_MESENCHYMAL_TRANSITION”, and “VEGF_A_UP.V1_DN”.

### Immune infiltration analysis

Immune score, stromal score, and estimate score representing immune infiltration, stromal cell level, and purity of tumor, respectively were obtained from Estimate website (https://bioinformatics.mdanderson.org/estimate/index.html) and compared between AVEN^high^ and AVEN^low^ patients. Furthermore, multiple deconvolution tools including Timer, CIBERSORT, EPIC, xCEll, Quanti-seq and MCP-counter (Li et al., 2017; Racle et al., 2017; Sturm et al., 2019) were utilized to examine various types of immune cells in tumor tissue. Immune infiltration data was obtained from Timer (https://cistrome.shinyapps.io/timer/) and was visualized by boxplot, using the R package via ggplot2.

### Prediction of immunotherapy response

To evaluate the prediction value of AVEN in immunotherapy response, Tumor Immune Dysfunction and Exclusion (TIDE) score (Jiang et al., 2018) was calculated (http://tide.dfci.harvard.edu/). Consequently, the expression levels of immune checkpoint genes and functional genes associated with cytotoxic T cells were analyzed in AVEN^high^ and AVEN^low^ LUAD. Furthermore, we extended our investigation to encompass additional immunotherapy response markers indicative of B cells, testing these markers in AVEN^high^ and AVEN^low^ LUAD patients.

### Establishment of an AVEN-derived prognostic genes model

To determine the correlation between each gene from the DEGs and the overall survival of LUAD patients, Univariate Cox Regression model was employed. AVEN-derived genes with *p*-value < 0.01 were regarded as AVEN-derived prognostic factors. Followed by Random Survival Forest analysis, the relative importance of each gene was calculated. Genes with relative importance >0.5 were used in the Multivariate Cox Regression model. Step forward Cox regression was utilized to optimize the model. The AVEN-derived genomic model was formulated as follows (Abd ElHafeez et al., 2021):

$$\begin{array}{ll} Risk\;score & =h0\left(t\right)\times \exp(KRT6A\times 0.0002919+SLC16A3\times 0.0045+CTSL\\ & \times 0.0008009+LDHA\times 0.007940+CDC42EP2\times 0.0147) \end{array}$$

The h0(t) represents the baseline hazard at time t, which denotes the hazard of an individual when all predictor variables are set to 0. Subsequently, based on the calculated risk scores, patients were categorized into high-risk and low-risk groups using the mean risk score. The survival and survminer packages were utilized to determine the survival state of LUAD patients in both the TCGA and GEO cohorts (GSE50081, GSE31210). ROC curves were generated to test the specificity and sensitivity of the AVEN-derived prognostic gene model by using the survivalROC package.

### Web-based bioinformatic analysis

PrognoScan (Mizuno et al., 2009) was employed to assess the prognostic significance of AVEN across multiple LUAD data cohorts. Additionally, AVEN protein abundance was examined using the cProSite (Wang et al., 2023).

### Statistical analysis

Statistical analysis was performed with R software (v4.2.1) and its suitable packages. In this study, group comparisons were performed using Student’s t-test, and the interaction between variables were examined using the Spearman correlation test.

## Result

### A high level of AVEN is associated with poor survival in LUAD

**FIGURE 1:**
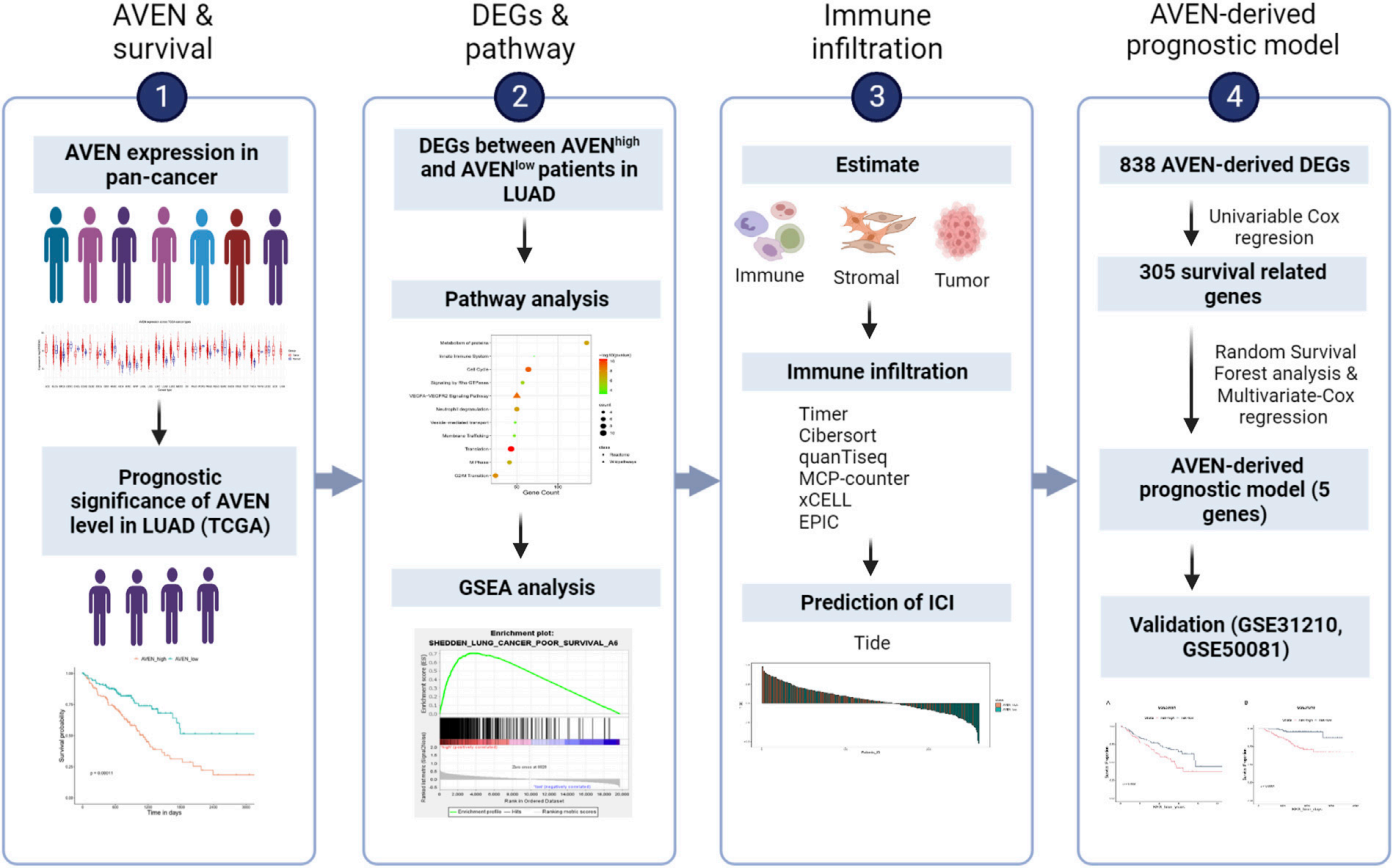
Brief overview of this study. LUAD: Lung adenocarcinoma. DEGs: Differentially expressed genes. TIDE: Tumor Immune Dysfunction and Exclusion. ICI: Immune checkpoint inhibitor.

**FIGURE 2:**
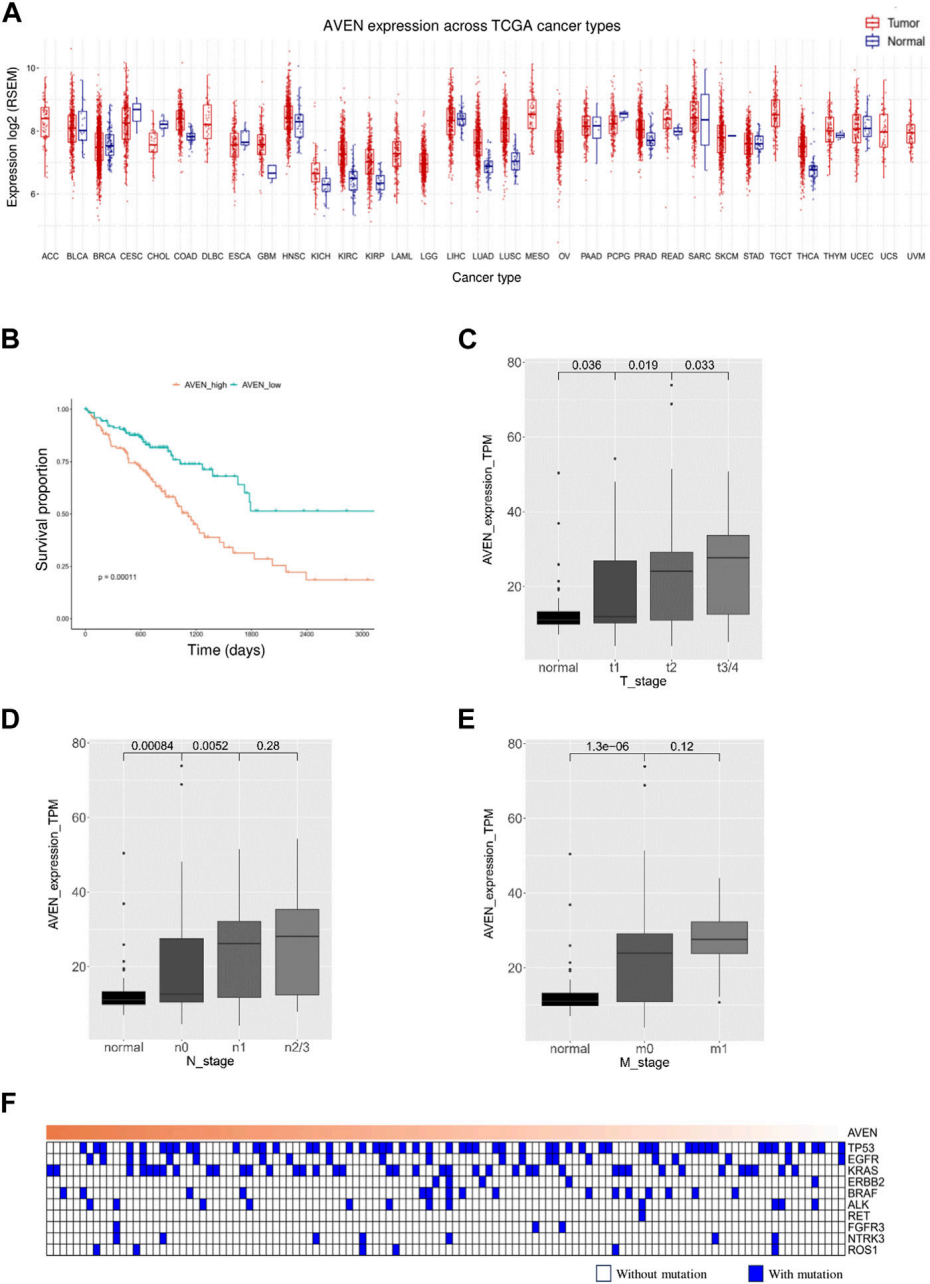
A high level of AVEN is associated with poor survival **(A)** mRNA level of AVEN in thirty-three different types of cancer and normal tissues. **(B)** Survival analysis in AVENhigh and AVENlow LUAD patients derived from TCGA database. **(C)** AVEN mRNA expression level in different T stages in LUAD and normal tissue. **(D)** AVEN mRNA expression level in different N stages in LUAD and normal tissue. **(E)** AVEN mRNA expression level in different M stages in LUAD and normal tissue. **(F)** Mutation landscape of driver genes associated with AVEN expression.

The role of AVEN in cancer is systematically studied according to the workflow shown in Figure 1. First, the GSCA web tool was exploited (Liu et al., 2023) to identify the AVEN mRNA level in various cancer types. AVEN was highly expressed in six types of cancer compared to normal tissue: colon adenoma (COAD), Kidney renal clear cell carcinoma (KIRC), Kidney renal papillary cell carcinoma (KIRP), Lung adenocarcinoma (LUAD), Lung squamous cell carcinoma (LUSC), and Thyroid carcinoma (THCA) (Figure 2A). On the other hand, a slight downregulation of AVEN was observed in two types of cancer, Cholangiocarcinoma (CHOL), and Cervical squamous cell carcinoma (CESC). Besides mRNA, AVEN protein level was analyzed using the cProSite database, which showed a higher AVEN protein abundance in Breast cancer, Colon cancer, Kidney cancer, Liver cancer, Lung adenocarcinoma, Lung squamous cell carcinoma, and ovarian cancer compared to its adjacent normal tissue (Supplementary Figure S1). Given our interest in the oncogenic role of AVEN, we performed GEPIA analysis to determine the overall survival in the six tumor types with elevated AVEN mRNA expression: COAD, KIRC, KIRP, LUAD, LUSC and THCA (Supplementary Figure S2). Notably, AVEN showed a significant prognostic effect only in LUAD. To further validate this finding and investigate the oncogenic features of AVEN, we acquired the mRNA sequencing data and clinical information of LUAD patients from the TCGA dataset. Based on the expression level of AVEN, LUAD patients were classified into two groups: AVEN^high^ (top 25%) and AVEN^low^ (bottom 25%), and overall survival was investigated using Kaplan-Meier analysis (Figure 2B). Consistent with GEPIA results, high AVEN expression was markedly associated with poor survival in LUAD. Before studying details of underlying molecular and cellular mechanism of AVEN, patient characteristics of AVEN^high^ and AVEN^low^ gruop were analyzed (Table 1). As shown in Table 1, AVEN expression did not show significant differences in age, race, and smoking but only in gender. Interestingly, the value of the T stage and N stage showed significant differences between the two groups. TNM classification is a system that defines tumor size (T), regional lymph amount (N), and spread of cancer (M) in a patient’s body (Rosen and Sapra, 2022). Thus, we wondered whether AVEN contributes to tumor progression according to TNM classification. Notably, AVEN showed a gradual increase with tumor progression when AVEN expression was seen in normal tissue and different T stages (Figure 2C). AVEN expression also increased in the N1 stage compared to N0 (Figure 2D). We further analyzed AVEN expression in different M stages, however, there were no significant differences in the number of patients in different M stages between AVEN^high^ and AVEN^low^ patients (Table 1). Consistently, AVEN expression was similar between M0 and M1 stages (Figure 2E). Since genetic alterations can lead to the activation of various signaling pathways that promote the growth and survival of cancer cells (Skoulidis and Heymach, 2019), we next examined genetic alterations in driver genes that were well defined in LUAD. However, no significant associations between AVEN expression and genetic mutations in EGFR, KRAS, ALK, ROS1, BRAF, *etc.*, Were observed (Figure 2F).

**TABLE 1:** Characteristics of AVEN^high^ and AVEN^low^ patients in LUAD.

	AVEN^high^ (N = 130)	AVEN^low^ (N = 130)	*p*
**Age (years)**	65.1 ± 9.7	64.5 ± 10.5	0.624
**T stage**			0.03
t1	31 (23.1%)	55 (41.9.0%)	
t2	75 (57.2%)	66 (50.3%)	
t3	15 (11.5%)	8 (6.1%)	
t4	9 (6.9%)	1 (0.8%)	
uncharacterized	1 (0.8%)	1 (0.8%)	
**N stage**			0.001
n0	67 (51.1%)	93 (71.5%)	
n1	33 (25.2%)	17 (13.1%)	
n2	27 (20.6%)	11 (8.5%)	
n3	0 (0.0%)	1 (0.8%)	
uncharacterized	4 (3.1%)	8 (6.2%)	
**M stage**			0.061
m0	92 (70.8%)	84 (65.6%)	
m1	11 (8.4%)	5 (3.8%)	
uncharacterized	27 (20.8%)	39 (30.5%)	
**Gender**			0.047
female	62 (47.3%)	79 (60.3%)	
male	69 (52.7%)	52 (39.7%)	
**Radiation therapy**			0.731
no	98 (83.8%)	106 (86.2%)	
yes	19 (16.2%)	17 (13.8%)	
**Race**			0.72
Asian	3 (2.7%)	3 (2.6%)	
black or African American	9 (8.0%)	13 (11.2%)	
white	100 (89.3%)	100 (86.2%)	
**AVEN expression (TPM)**	31.9 ± 8.5	10.5 ± 2.0	0
**Smoke**			0.788
no	90 (68.7%)	93 (71.0%)	
yes	41 (31.3%)	38 (29.0%)	

### Functional signaling pathways associated with AVEN

**FIGURE 3:**
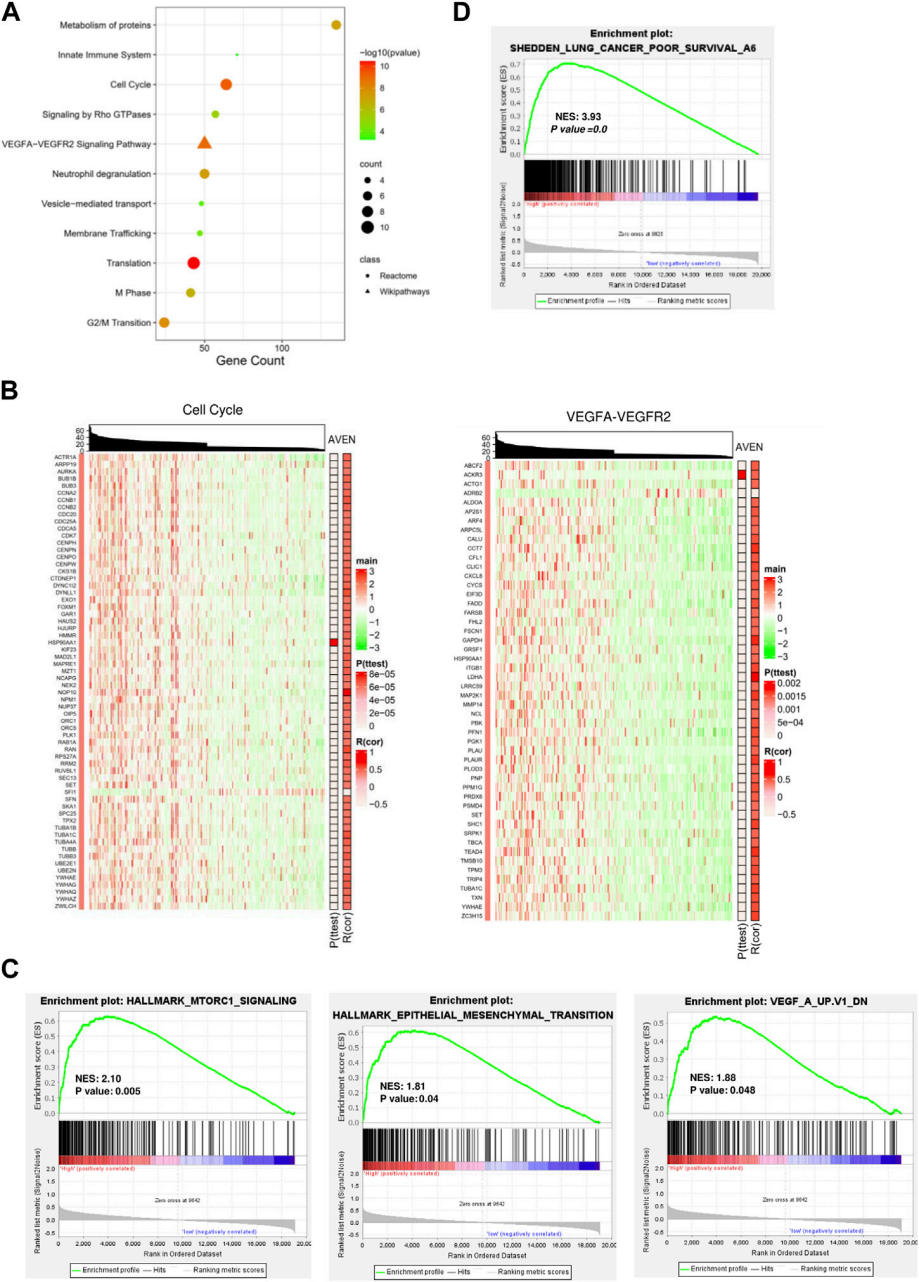
Functional signaling pathways associated with AVEN expression. **(A)** Pathway enrichment analysis with DEGs. **(B)** Heatmaps visualized the association of AVEN expression with genes involved in cell cycle and VEGF-VEGFR2 pathways. **(C)** GSEA analysis in AVENhigh and AVENlow groups using MTORC1, Epithelial-mesenchymal-transition (EMT), and VEGF signature. **(D)** GSEA analysis in AVENhigh and AVENlow groups using a lung cancer poor survival signature.

We next investigated molecular and cellular pathways associated with AVEN. First, DEGs between AVEN^high^ and AVEN^low^ groups were analyzed by using Spearman’s rank correlation test with the criteria of the absolute R-value over 0.4. A total of 838 genes were obtained (Supplementary Table S1), followed by pathway enrichment analysis via the ConsensusPathDB website tool. We found that those DEGs were mainly involved in the biological process pathways (such as metabolism of proteins, and membrane trafficking), oncogenic pathways (such as the cell cycle and VEGFA-VEGFR2 Signaling Pathway), and immune regulation process (such as neutrophil degranulation, and innate immune system) (Figure 3A).

Given the fact that VEGF plays an important role in tumor progression and angiogenesis (Jiang et al., 2020), we suggested that AVEN might enhance tumor aggressiveness by promoting the cell cycle and angiogenesis. To better demonstrate a correlation between AVEN and the gene expression profile in the cell cycle or VEGFA-VEGFR2 Signaling Pathway, heatmaps were introduced for visualization. Genes in these pathways were distinctly upregulated by AVEN overexpression (Figure 3B). Gene set enriched analysis (GSEA) was applied to further investigate the oncogenic role of AVEN. Genes in MTORC1 signaling, epithelial-mesenchymal-transition (EMT), and VEGF pathways were remarkably enriched in AVEN^high^ patients (Figure 3C). MTOR pathway is a key pathway to regulate cell growth. EMT and VEGFA also play a critical role in tumor progression (Brabletz et al., 2018; Zou et al., 2020). Furthermore, we found that AVEN overexpression was associated with a poor survival genomic signature (Figure 3D) in NSCLC (NES = 3.43, *p*-value = 0.0), consistent with results shown in Figure 2B.

### AVEN-associated immune infiltration landscape in LUAD

The tumor microenvironment (TME) consists of various cells such as normal epithelial, vascular cells, stromal cells, and immune cells. Immune cells, such as T cells, B cells, natural killer cells, dendritic cells, macrophages, and others are attracted to the site of a tumor by various signals, including chemokines and cytokines produced by tumor cells and stromal cells and regulate tumor cells proliferation/apoptosis (Galli et al., 2020). Considering our finding of AVEN-associated signaling pathways such as innate immune system, neutrophil degranulation, and EMT (Figures 3A, C), it suggested unique TME features associated with AVEN expression.

**FIGURE 4:**
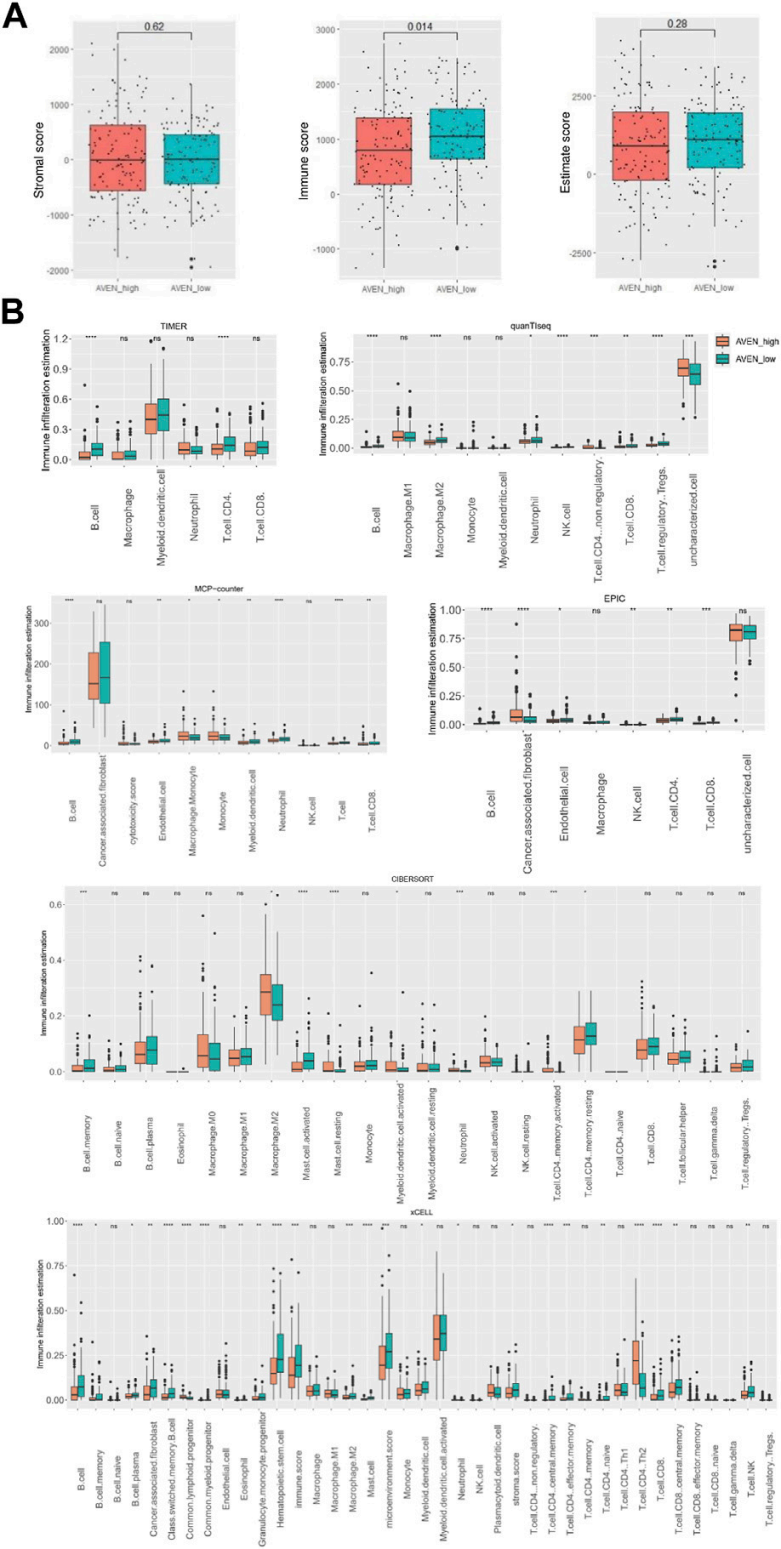
Immune infiltration landscape associated with AVEN. **(A)** Stromal score, immune score and estimate score in AVENhigh and AVENlow groups. **(B)** immune infiltration evaluation by diverse deconvolution tools including TIMER, MCP-Counter, EPIC, quanTiseq, xCELL, and CIBERSORT.

Thus, we first analyzed immune cells and stromal cells using the ESTIMATE algorithm in AVEN^high^ and AVEN^low^ groups. Despite the fact that there was no significant difference in stromal score, AVEN^high^ patients showed lower immune scores compared to AVEN^low^ patients, which suggests the functional possibility of AVEN to inhibit the abundance of immune cells in the TME. Based on the stromal score and immune score, the Estimate score was calculated to infer the purity of tumor tissue (Yoshihara et al., 2013), and the result showed AVEN^high^ and AVEN^low^ groups shared a similar tumor purity (Figure 4A). To explore the diverse types of immune cells, multiple deconvolution tools were employed, including Timer, CIBERSORT, EPIC, xCEll, Quanti-seq, and MCP-counter (Sturm et al., 2019). These tools utilize different methodologies to analyze the RNA-seq data and provide insights into the composition and abundance of immune cell populations (Im and Kim, 2023). The analysis using various deconvolution tools revealed that B cells were significantly more abundant in AVEN^low^ groups. However, different patterns were observed for other immune cell types depending on the specific tool used. For instance, Timer and EPIC indicated that CD4 T cells were significantly higher in AVEN^low^ groups, while Quanti-Seq suggested higher levels in AVEN^high^ groups. Additionally, CIBERSORT analysis showed that activating memory CD4 T cells were more abundant in AVEN^high^ groups, whereas resting memory CD4 T cells were higher in AVEN^low^ groups (Figure 4B). EPIC, MCP-counter and xCEll subtracted cancer associated fibroblast (CAF) from tumor. Specifically, EPIC and MCP-counter indicated that CAF were significantly more abundant in AVEN^high^ groups. However, in contrast to these findings, xCEll tool showed lower levels of CAF in AVEN^high^ groups (Figure 4B).

### A high level of AVEN expression predicts a poor immunotherapy response

**FIGURE 5:**
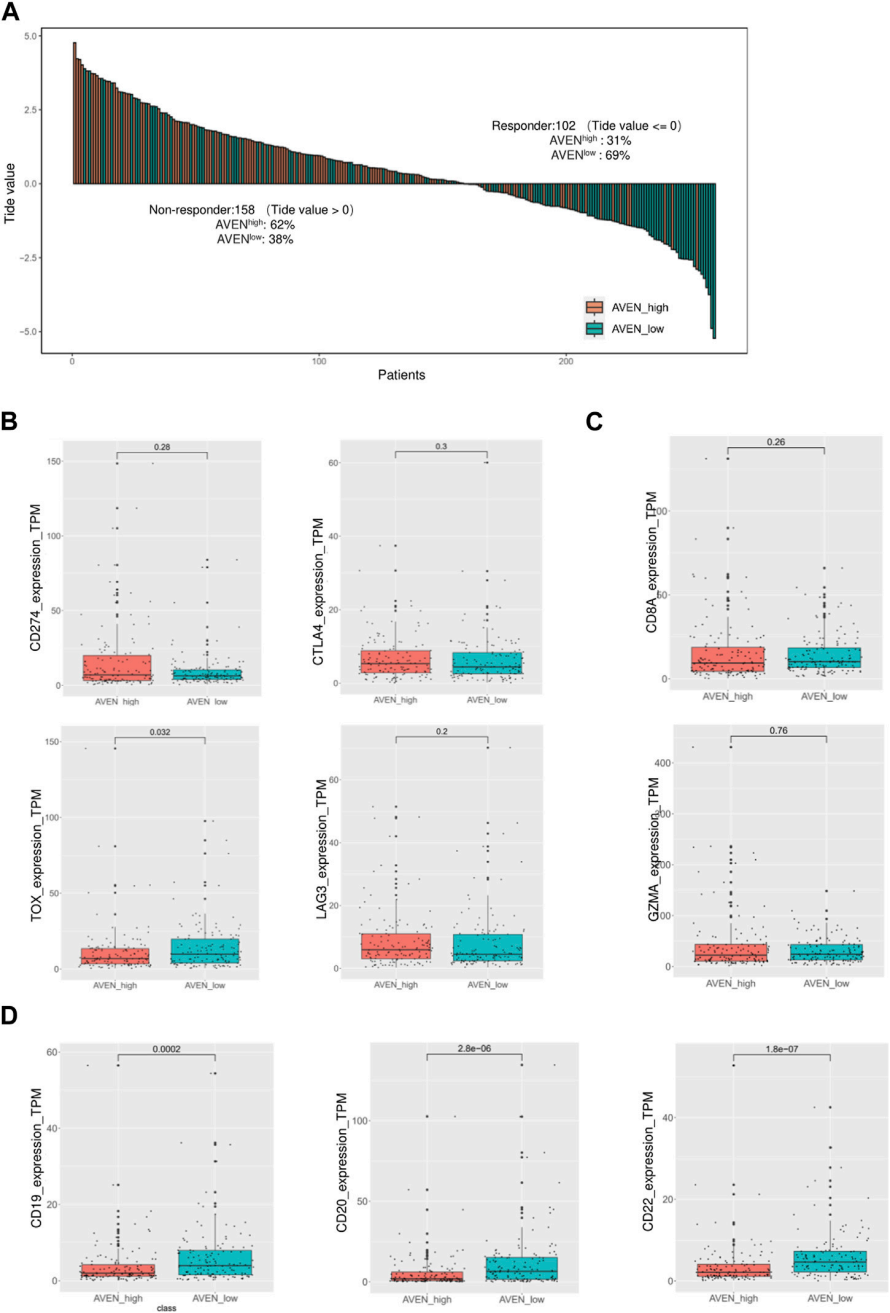
Immunotherapy response prediction based on AVEN expression. **(A)** Bar-plot of TIDE score among 260 patients with different AVEN expression levels. **(B)** Expression of immune checkpoint genes in AVENhigh and AVENlow patients. **(C)** Genes expression related to the cytotoxicity activity of tumor-killing T cells in AVENhigh and AVENlow patients. **(D)** mRNA expression of B cell markers was compared between AVEN^high^ and AVEN^low^ patients.

Immune checkpoint inhibitors (ICIs) in cancer treatments have demonstrated significant potential in enhancing antitumor immune responses and improving patient outcomes (Bondhopadhyay et al., 2020; Robert, 2020). However, their responsiveness remains limited, necessitating the identification of predictive biomarkers (Bai et al., 2020; Błach et al., 2021). Therefore, we investigated whether AVEN could serve as a predictive biomarker for ICIs response using the TIDE algorithm, in addition to assessing the expression of immune checkpoint genes. To explore the AVEN-associated immunotherapy response, RNA-seq data from 260 patients in AVEN^high^ and AVEN^low^ groups were processed in TIDE tool. We found that among the 158 non-responders, 62% (n = 98) belonged to the AVEN^high^ group, while 38% (n = 60) were in the AVEN^low^ group. Specifically, among the AVEN^high^ patients (n = 130), only 24% (n = 32) were predicted to be responders based on the TIDE tool (Figure 5A, Supplementary Table S2). Subsequently, expression of immune checkpoint genes was examined. Immune checkpoint genes such as CD274, CTLA4, and LAG3 (Qin et al., 2019; Wang et al., 2019; Hu et al., 2021) had no significant differences between AVEN^high^ and AVEN^low^ groups (Figure 5B). Genes associated with anti-tumor CD8 T cells such as CD8A and GZMA (van der Leun et al., 2020) were also examined, but no differences were observed (Figure 5C). Immune checkpoints and CD8 T cell function are regarded as the essential indicators for immunotherapy respond, however the immunotherapy resistance is far more complicated than that. For example, a lower mutation burden (TMB), dysfunctional MHCs complex, or immunosuppressive tumor microenvironment (Lei et al., 2020) can result in a lower responsiveness in immunotherapy treatment without altering the expression level of immune checkpoint and CD8 T cell markers. Moreover, in our current study, the observation of diminished B cell infiltration (Figure 4B) in AVEN^high^ patients have spurred us to propose additional possibilities that B cell mediates immunotherapy resistance. With the knowledge that B cells also express the receptors of PD1/PDL1/CTLA4 (Kim et al., 2021) and have been established as a favorable prognostic marker in NSCLC (Germain et al., 2014), there exists a theoretical basis for B cells to respond to immune checkpoint inhibitors. We extended our analysis to assess the expression of B cell markers in LUAD. As depicted in Figure 5D, there was a significant reduction in mRNA levels of CD19, CD20, and CD22 in patients with elevated AVEN expression, further suggesting a distinct immune phenotype in AVEN^high^ patients, characterized by reduced B cell infiltration.

### Developing an AVEN-derived genomic model for LUAD prognosis

**FIGURE 6:**
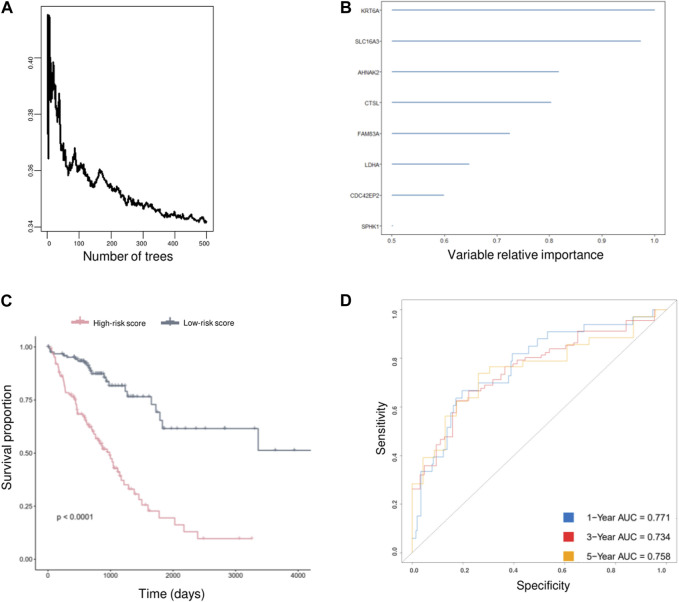
AVEN-derived prognostic model in LUAD. **(A)** Random survival forest analysis with DEGs. **(B)** AVEN-derived genes with a relative importance value over 0.5. **(C)** Survival analysis between LUAD patients with high-risk score and low-risk score using TCGA LUAD data. **(D)** Time-dependent ROC curves based on optimized AVEN-derived prognostic model.

As AVEN displayed significant predictive value in terms of LUAD survival within the TCGA database, we were motivated to investigate its prognostic utility across other cohorts. Employing PrognoScan, we delved into the influence of AVEN expression on different datasets. While a substantial correlation with LUAD survival was observed in some cohorts, it is important to note that certain cohorts exhibited limitations in significant prognostic effect of AVEN (Supplementary Table S3). Since AVEN-derived DEGs showed remarkable effect in pivotal oncogenic pathways, we further investigated the prognosis effect of 838 DEGs by using Univariate Cox Regression (Ma et al., 2022; Yu et al., 2022). 305 of survival-related genes with *p*-value < 0.01 (Supplementary Table S4) were shown to contribute to LUAD patient survival and determined as prognostic factors, which were then used as input for the Random Survival Forest analysis (Figure 6A). Following the Random Survival Forest analysis, the relative importance of the above prognostic genes was calculated and ranked. Eight genes, KRT6A, SLC16A3, AHNAK2, CTSL, FAM83A, LDHA, CDC42EP2, and SPHK1, were selected with a relative importance value of over 0.5 (Figure 6B), and Multivariate-Cox regression model was developed with those genes. In order to optimize survival prediction model, step-forward Multivariate Cox analysis was used to further screen the valuable prognostic genes containing KRT6A, SLC16A3, CTSL, LDHA, and CDC42EP2. According to the model, the risk score of each patient was identified as follows: $h0\left(t\right)\times \exp\left(KRT6A\times 0.0002919+SLC16A3\times 0.0045+CTSL\times 0.0008009+LDHA\times 0.007940+CDC42EP2\times 0.0147\right)$ . In accordance with the mean risk score, LUAD patients were classified into two subpopulations, high and low risk score. Kaplan-Meier survival analysis showed LUAD individuals with low-risk scores had a greater benefit in survival compared with a high-risk score population, which revealed that the AVEN-derived prognostic model was able to estimate patients’ prognosis according to risk score (Figure 6C). Additionally, the time-dependent ROC curves were performed and confirmed that AVEN derived prognostic model showed potency to predict patient prognosis (Figure 6D).

### Validation of AVEN-derived prognostic model in LUAD

**FIGURE 7:**
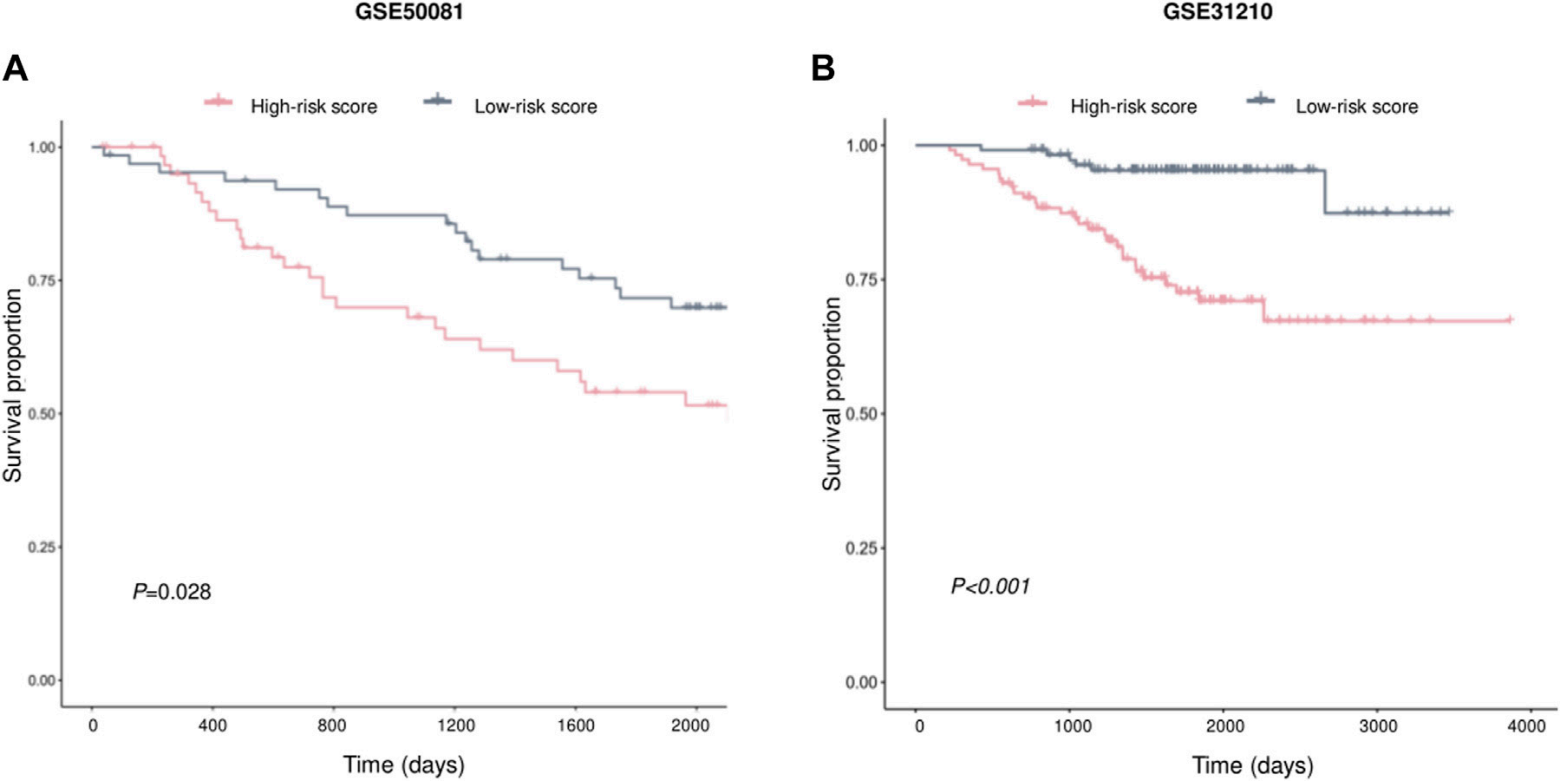
Validation of prognostic effect of AVEN-derived prognostic model in LUAD. **(A)** External validation of AVEN-derived prognostic model using GSE50081. **(B)** External validation of AVEN-derived prognostic model using GSE31210.

Two independent external datasets, GSE50081 and GSE31210, were used to evaluate AVEN-derived prognostic model. As shown in Figures 7A, B, LUAD patients with low-risk score had better survival, which indicated that the AVEN-derived prognostic model is effective and sufficient in predicting LUAD survival.

## Discussion

Even though AVEN has been found as an apoptosis, caspase activation inhibitor, its role in solid tumor including lung cancer was rarely studied. To analyze the role of AVEN systematically, we examined AVEN mRNA in 33 cancer types and found high AVEN expression in COAD, KIRC, KIRP, LUAD, LUSC and THCA compared to normal tissue, but interestingly high AVEN expression was associated with poor survival only in LUAD (Supplementary Figure S2). The impact of AVEN on tumor progression was analyzed in the LUAD patients grouped by the TNM classification, and we found that AVEN expression was significantly associated with the tumor burden of the main tumor and the number of lymph nodes contained in cancer (Figures 2C–E). Specifically, AVEN expression was positively correlated with the size and/or extent of the main tumor, suggesting the oncogenic role of AVEN. Before delving into the cellular and molecular events in AVEN-associated LUAD, we questioned how AVEN is upregulated in LUAD. Interestingly, we found that AVEN upregulation in LUAD was not due to genomic amplification (Supplementary Table S5). Although it is unclear which factor regulates AVEN expression in the cancer cells in the absence of genomic amplification, previous studies have suggested potential regulatory mechanism. MiR-30 family (Ouzounova et al., 2013) has been reported to negatively regulate the AVEN expression in the breast cancer cell line, while Foxo1 (Cai et al., 2017) has a positive regulatory effect on AVEN expression in regulatory T cells. Hence, we propose that AVEN overexpression in cancer cells may result from the interplay of miRNA, transcription factor, and epigenetic modifications.

We furthermore analyzed signaling pathways altered by AVEN expression to understand the underlying mechanism of AVEN in tumor and tumor microenvironment. First, 838 DEGs were obtained between AVEN^high^ and AVEN^low^ groups by using Spearman’s rank correlation test using the criteria of absolute R-value over 0.4, followed by pathway enrichment analysis (Figure 3A). The results were explained by three different functional cellular pathways, biological process pathway with metabolism of proteins and membrane trafficking, oncogenic pathways with cell cycle and VEGFA-VEGFR2 signaling pathway, and immune regulation process with neutrophil degranulation and innate immune system.

The majority of genes under cell cycle and VEGFA-VEGFR2 pathways were dramatically upregulated in AVEN^high^ patients (Figure 3B). Although AVEN is known as an apoptosis inhibitor, further studies are needed to confirm whether AVEN boosts the cell cycle in cancer by inhibiting apoptosis. Apart from losing control of cell division, cancer cells have the ability to use various immune escape mechanisms from our immune surveillance, such as migrating to other parts of the organs via the blood vessel. One of the critical signaling pathways involved in angiogenesis is the VEGFA-VEGFR2 pathway. Activation of the VEGFA-VEGFR2 pathway is commonly observed in many types of cancer and is associated with poor prognosis. Furthermore, GSEA analysis also showed that AVEN^high^ LUAD patients have more active oncogenic pathways including mTOR signaling (Figure 3C). These oncogenic pathways are targeted by drugs such as Palbociclib (CDK4/6 inhibitors) (Mills et al., 2017), Aflibercept (anti-VEGF agent) (Zirlik and Duyster, 2018) and Rapamycin (mTOR signaling) (*Rapamycin hits the target | Nature Reviews Cancer*, no date), all of which are in a clinical use. This further highlights the potential significance of AVEN as a promising therapeutic target in cancer treatment.

As tumor and their neighboring cells such as fibroblast and immune cells receive and send proliferation/apoptosis signals from each other (Galli et al., 2020), we examined the immune and stromal scores using ESTIMATE algorithm. AVEN^high^ patients showed lower immune scores compared to AVEN^low^ patients, while stromal scores had no significant difference (Figure 4A). In order to have a better understanding of immune cell infiltration correlated with AVEN, Timer, CIBERSORT, EPIC, xCEll, Quanti-seq, and MCP-counter were exploited to study the infiltration of diverse cell types (Figure 4B). These tools are available to analyze infiltrated immune cells using bulk RNA-seq, and each tool has been developed using a different methodology. B cells were significantly lower in AVEN^high^ expressing group by all the algorithms indicating a weakened anti-tumor immunity (Figure 4B). The compromised B cell phenotype in AVEN^high^ patients was further confirmed by examining expression level of B cells marker such as CD19, CD20 and CD22 (Figure 5D). B cell belongs to antigen-presenting cells (APC), which is able to gain, process, and present tumor-associated antigens for T cells activation (*Nature Reviews Immunology*, no date). In one study, the depletion of B cells decreased the production of type I T cells (Th1) and caused scarcity of IFN-γ, supporting the crucial role that B cells may play in T cell immunity (DiLillo et al., 2010). In addition to their role in facilitating T cell activation, B cells also play a pivotal role in the tumor microenvironment, such as tumor-specific antibodies mediated antibody-dependent cell cytotoxicity (ADCC) (Germain et al., 2014; Sarvaria et al., 2017), complement activation (Dunkelberger and Song, 2010), and the formation and maintenance of tertiary lymphoid structures (TLS). Furthermore, independent cohort studies have highlighted the association of B cells with immune therapy response (Cabrita et al., 2020; Helmink et al., 2020; Petitprez et al., 2020). Therefore, even though no difference was observed in CD8 T cell markers (Figure 5C), the AVEN-mediated downregulation of B cell could potentially contribute to insufficient immunity, possibly resulting in an immunotherapy resistance phenotype through mechanisms that bypass T cell. This observation is further supported by the result from TIDE tool, indicating the association of AVEN in immunotherapy resistant effects (Figure 5A). Besides, CAF was upregulated in AVEN^high^ patients as shown by EPIC and MCP-counter even though xCELL showed the opposite results (Figure 4B). CAF is involved in the matrix remodeling process and soluble factor secretion including VEGF, Exosomes, HGF, etc., which are the underlying mechanisms of tumor metastasis (Sarvaria et al., 2017). Because of the fundamental role of CAF in tumor progression, the correlation between AVEN and CAF needs to be further investigated.

In recent years, many prognostic biomarkers have been reported, however their applications are often limited due to the variations observed between different cohorts. Similarly, AVEN demonstrated a notable correlation with LUAD survival in some cohorts, but its significant prognostic effects were limited (Supplementary Table S3). To address the limitations associated with relying on a single prognostic marker, we developed a more comprehensive approach. We focused on the identification of AVEN-related genes and their association with patient survival. Initially, we identified 838 DEG that exhibited a correlation with AVEN expression. Subsequently, we performed Univariate Cox analysis, filtering down to 305 survival-related genes that displayed significant prognostic effects in LUAD patients within the TCGA cohort. To further refine our prognostic model, we employed Random Survival Forest analysis and Multiple Cox Regression analysis. The resulting model, consisting of five AVEN-related genes, demonstrated robust predictive capabilities for patient survival in both the TCGA cohort and two separate GEO cohorts, highlighting the potential of our AVEN-derived prognostic model as a valuable tool for LUAD prognosis and offering enhanced accuracy and applicability across diverse datasets.

Further investigation was conducted to explore the relationship between the identified five genes (KRT6A, SLC16A3, CTSL, LDHA, CDC42EP2) and AVEN expression. Supplementary Table S6 revealed a high correlation between these genes and AVEN expression. However, in-depth analysis using Protein-Protein Interaction (PPI) network analysis did not uncover any direct interactions among the proteins encoded by these genes (Supplementary Figure S3). It is worth noting that the lack of observed interactions in the PPI network may be attributed to insufficient available information or limitations in the current understanding of these interactions. Further studies are warranted to delve into this aspect and gather more comprehensive data in the future. In addition, previous studies have identified Bcl-xl and Apaf-1 as AVEN interacting proteins. These interactions are known to retain Bcl-xl mediated anti-apoptotic activity and prevent Apaf-1 mediated caspase activation (Chau et al., 2000). However, in this study, we observed that the expressions of Bcl-xl and Apaf-1 do not show significant association with AVEN expression (Supplementary Table S6). In our efforts to predict the underlying molecular mechanisms of AVEN, the results from this study suggest that AVEN may exert control over oncogenic events through mechanisms involving cell cycle regulation, VEGFR pathway. mTOR signaling, EMT and immune pathways. However, to gain a deeper understanding of molecular mechanism underlying AVEN’s involvement in LUAD, further investigations are imperative to elucidate the specific molecular details associated with AVEN’s function in the oncogenic process in LUAD.

In summary, our study elucidated the significance of AVEN in LUAD, showing its association with poor survival and its potential role in tumor progression and immune evasion. We developed an AVEN‐derived prognostic model, incorporating five AVEN‐related genes, which exhibited robust predictive capabilities for patient survival in diverse cohorts, highlighting the potential of our AVEN‐derived prognostic model as a valuable tool for LUAD prognosis.

## Data Availability

The datasets presented in this study can be found in online repositories. The names of the repository/repositories and accession number(s) can be found in the article/Supplementary Material.

## References

[B1] Abd ElHafeez S. D'Arrigo G. Leonardis D. Fusaro M. Tripepi G. Roumeliotis S. (2021). Methods to analyze time-to-event data: the Cox regression analysis’. Oxidative Med. Cell. Longev. 2021, 1302811. Available at:. 10.1155/2021/1302811 PMC8651375 34887996

[B2] Bai R. Lv Z. Xu D. Cui J. (2020). Predictive biomarkers for cancer immunotherapy with immune checkpoint inhibitors. Biomark. Res. 8 (1), 34. Available at:. 10.1186/s40364-020-00209-0 32864131 PMC7450548

[B3] Błach J. Wojas-Krawczyk K. Nicoś M. Krawczyk P. (2021). Failure of immunotherapy-the molecular and immunological origin of immunotherapy resistance in lung cancer. Int. J. Mol. Sci. 22 (16), 9030. Available at:. 10.3390/ijms22169030 34445735 PMC8396490

[B4] Bondhopadhyay B. Sisodiya S. Chikara A. Khan A. Tanwar P. Afroze D. (2020). Cancer immunotherapy: a promising dawn in cancer research. Am. J. Blood Res. 10 (6), 375–385. 33489447 PMC7811907

[B5] Brabletz T. Kalluri R. Nieto M. A. Weinberg R. A. (2018). EMT in cancer. Nat. Rev. Cancer 18 (2), 128–134. Available at:. 10.1038/nrc.2017.118 29326430

[B6] Cabrita R. Lauss M. Sanna A. Donia M. Skaarup Larsen M. Mitra S. (2020). Tertiary lymphoid structures improve immunotherapy and survival in melanoma. Nature 577 (7791), 561–565. Available at:. 10.1038/s41586-019-1914-8 31942071

[B7] Cai Z. Liu H. Wu X. (2017). Forkhead-box transcription factor 1 affects the apoptosis of natural regulatory T cells by controlling Aven expression. BMC Immunol. 18 (1), 16. Available at:. 10.1186/s12865-017-0198-8 28283017 PMC5345239

[B8] Cerami E. Gao J. Dogrusoz U. Gross B. E. Sumer S. O. Aksoy B. A. (2012). The cBio cancer genomics portal: an open platform for exploring multidimensional cancer genomics data. Cancer Discov. 2 (5), 401–404. Available at:. 10.1158/2159-8290.CD-12-0095 22588877 PMC3956037

[B9] Chan B. A. Hughes B. G. M. (2015). Targeted therapy for non-small cell lung cancer: current standards and the promise of the future. Transl. Lung Cancer Res. 4 (1), 36–54. Available at:. 10.3978/j.issn.2218-6751.2014.05.01 25806345 PMC4367711

[B10] Chau B. N. Cheng E. H. Kerr D. A. Hardwick J. M. (2000). Aven, a novel inhibitor of caspase activation, binds bcl-xL and apaf-1. Mol. Cell. 6 (1), 31–40. Available at:. 10.1016/S1097-2765(05)00021-3 10949025

[B11] Choi J. Hwang Y. K. Sung K. W. Kim D. H. Yoo K. H. Jung H. L. (2006). Aven overexpression: association with poor prognosis in childhood acute lymphoblastic leukemia. Leukemia Res. 30 (8), 1019–1025. Available at:. 10.1016/j.leukres.2005.11.001 16388850

[B12] Dendritic cells in cancer immunology (2023). Dendritic cells in cancer immunology and immunotherapy | Nature Reviews Immunology. Available at: https://www.nature.com/articles/s41577-019-0210-z (Accessed: March 20, 2023).

[B13] DiLillo D. J. Yanaba K. Tedder T. F. (2010). B cells are required for optimal CD4+ and CD8+ T cell tumor immunity: therapeutic B cell depletion enhances B16 melanoma growth in mice. J. Immunol. 184 (7), 4006–4016. Available at:. 10.4049/jimmunol.0903009 20194720 PMC3733120

[B14] Dunkelberger J. R. Song W.-C. (2010). Complement and its role in innate and adaptive immune responses. Cell. Res. 20 (1), 34–50. Available at:. 10.1038/cr.2009.139 20010915

[B15] Eißmann M. Melzer I. M. Fernández S. B. M. Michel G. Hrabě de Angelis M. Hoefler G. (2013). Overexpression of the anti-apoptotic protein AVEN contributes to increased malignancy in hematopoietic neoplasms. Oncogene 32 (20), 2586–2591. Available at:. 10.1038/onc.2012.263 22751129

[B16] Galli F. Aguilera J. V. Palermo B. Markovic S. N. Nisticò P. Signore A. (2020). Relevance of immune cell and tumor microenvironment imaging in the new era of immunotherapy. J. Exp. Clin. Cancer Res. 39 (1), 89. Available at:. 10.1186/s13046-020-01586-y 32423420 PMC7236372

[B17] Gao J. Aksoy B. A. Dogrusoz U. Dresdner G. Gross B. Sumer S. O. (2013). Integrative analysis of complex cancer genomics and clinical profiles using the cBioPortal. Sci. Signal. 6 (269), pl1. Available at:. 10.1126/scisignal.2004088 23550210 PMC4160307

[B18] Gelatti A. Drilon A. Santini F. C. (2019). Optimizing the sequencing of tyrosine kinase inhibitors (TKIs) in epidermal growth factor receptor (EGFR) mutation-positive non-small cell lung cancer (NSCLC). Lung cancer 137, 113–122. Available at:. 10.1016/j.lungcan.2019.09.017 31568888 PMC7478849

[B19] Germain C. Gnjatic S. Tamzalit F. Knockaert S. Remark R. Goc J. (2014). Presence of B cells in tertiary lymphoid structures is associated with a protective immunity in patients with lung cancer. Am. J. Respir. Crit. Care Med. 189 (7), 832–844. Available at:. 10.1164/rccm.201309-1611OC 24484236

[B20] Helmink B. A. Reddy S. M. Gao J. Zhang S. Basar R. Thakur R. (2020). B cells and tertiary lymphoid structures promote immunotherapy response. Nature 577 (7791), 549–555. Available at:. 10.1038/s41586-019-1922-8 31942075 PMC8762581

[B21] Hu F.-F. Liu C. J. Liu L. L. Zhang Q. Guo A. Y. (2021). Expression profile of immune checkpoint genes and their roles in predicting immunotherapy response. Briefings Bioinforma. 22 (3), bbaa176. Available at:. 10.1093/bib/bbaa176 32814346

[B22] Im Y. Kim Y. (2023). A comprehensive overview of RNA deconvolution methods and their application. Mol. Cells 46 (2), 99–105. Available at:. 10.14348/molcells.2023.2178 36859474 PMC9982058

[B23] Jiang P. Gu S. Pan D. Fu J. Sahu A. Hu X. (2018). Signatures of T cell dysfunction and exclusion predict cancer immunotherapy response. Nat. Med. 24 (10), 1550–1558. Available at:. 10.1038/s41591-018-0136-1 30127393 PMC6487502

[B24] Jiang X. Wang J. Deng X. Xiong F. Zhang S. Gong Z. (2020). The role of microenvironment in tumor angiogenesis. J. Exp. Clin. Cancer Res. 39 (1), 204. Available at:. 10.1186/s13046-020-01709-5 32993787 PMC7526376

[B25] Kamburov A. Wierling C. Lehrach H. Herwig R. (2009) ‘ConsensusPathDB—a database for integrating human functional interaction networks’, Nucleic Acids Res., 37(Database issue), pp. D623, D628. 10.1093/nar/gkn698 18940869 PMC2686562

[B26] Kim S. S. Sumner W. A. Miyauchi S. Cohen E. E. W. Califano J. A. Sharabi A. B. (2021). Role of B Cells in responses to checkpoint blockade immunotherapy and overall survival of cancer patients. Clin. Cancer Res. 27 (22), 6075–6082. Available at:. 10.1158/1078-0432.CCR-21-0697 34230025 PMC8976464

[B27] Lei Q. Wang D. Sun K. Wang L. Zhang Y. (2020). Resistance mechanisms of anti-PD1/PDL1 therapy in solid tumors. Front. Cell. Dev. Biol. 8, 672. 10.3389/fcell.2020.00672 32793604 PMC7385189

[B28] Li C. Tang Z. Zhang W. Ye Z. Liu F. (2021). GEPIA2021: integrating multiple deconvolution-based analysis into GEPIA. Nucleic Acids Res. 49 (1), W242–W246. Available at:. 10.1093/nar/gkab418 34050758 PMC8262695

[B29] Li T. Fan J. Wang B. Traugh N. Chen Q. Liu J. S. (2017). TIMER: a web server for comprehensive analysis of tumor-infiltrating immune cells. Cancer Res. 77 (21), e108–e110. Available at:. 10.1158/0008-5472.CAN-17-0307 29092952 PMC6042652

[B30] Liu C.-J. Hu F. F. Xie G. Y. Miao Y. R. Li X. W. Zeng Y. (2023). GSCA: an integrated platform for gene set cancer analysis at genomic, pharmacogenomic and immunogenomic levels. Briefings Bioinforma. 24 (1), bbac558. Available at:. 10.1093/bib/bbac558 36549921

[B31] Ma B. Wang K. Liang Y. Meng Q. Li Y. (2022). Molecular characteristics, oncogenic roles, and relevant immune and pharmacogenomic features of EVA1B in colorectal cancer. Front. Immunol. 13, 809837. 10.3389/fimmu.2022.809837 35250982 PMC8888821

[B32] Melzer I. M. Fernández S. B. M. Bösser S. Lohrig K. Lewandrowski U. Wolters D. (2012). The Apaf-1-binding protein Aven is cleaved by Cathepsin D to unleash its anti-apoptotic potential. Cell. Death Differ. 19 (9), 1435–1445. Available at:. 10.1038/cdd.2012.17 22388353 PMC3422468

[B33] Mills C. C. Kolb E. A. Sampson V. B. (2017). Recent advances of cell-cycle inhibitor therapies for pediatric cancer. Cancer Res. 77 (23), 6489–6498. Available at:. 10.1158/0008-5472.CAN-17-2066 29097609 PMC5712276

[B34] Mizuno H. Kitada K. Nakai K. Sarai A. (2009). PrognoScan: a new database for meta-analysis of the prognostic value of genes. BMC Med. Genomics 2, 18. Available at:. 10.1186/1755-8794-2-18 19393097 PMC2689870

[B35] Ouzounova M. Vuong T. Ancey P. B. Ferrand M. Durand G. Le-Calvez Kelm F. (2013). MicroRNA miR-30 family regulates non-attachment growth of breast cancer cells. BMC Genomics 14 (1), 139. Available at:. 10.1186/1471-2164-14-139 23445407 PMC3602027

[B36] Petitprez F. de Reyniès A. Keung E. Z. Chen T. W. W. Sun C. M. Calderaro J. (2020). B cells are associated with survival and immunotherapy response in sarcoma. Nature 577 (7791), 556–560. Available at:. 10.1038/s41586-019-1906-8 31942077

[B37] Qin S. Xu L. Yi M. Yu S. Wu K. Luo S. (2019). Novel immune checkpoint targets: moving beyond PD-1 and CTLA-4. Mol. Cancer 18 (1), 155. Available at:. 10.1186/s12943-019-1091-2 31690319 PMC6833286

[B38] Racle J. (2017). “Simultaneous enumeration of cancer and immune cell types from bulk tumor gene expression data,”eLife, e26476. Editor Valencia A., 6. Available at:. 10.7554/eLife.26476 29130882 PMC5718706

[B39] Rapamycin hits the target (2023). Rapamycin hits the target | nature Reviews cancer. Available at: https://www.nature.com/articles/nrc2341 (Accessed: September 24, 2023).

[B40] Robert C. (2020). A decade of immune-checkpoint inhibitors in cancer therapy. Nat. Commun. 11 (1), 3801. Available at:. 10.1038/s41467-020-17670-y 32732879 PMC7393098

[B41] Rosen R. D. Sapra A. (2022). TNM classification’, in *StatPearls*. Treasure island (FL): statPearls publishing. Available at: http://www.ncbi.nlm.nih.gov/books/NBK553187/ (Accessed: March 20, 2023). 31985980

[B42] Sarvaria A. Madrigal J. A. Saudemont A. (2017). B cell regulation in cancer and anti-tumor immunity. Cell. Mol. Immunol. 14 (8), 662–674. Available at:. 10.1038/cmi.2017.35 28626234 PMC5549607

[B43] Skoulidis F. Heymach J. V. (2019). Co-occurring genomic alterations in non-small-cell lung cancer biology and therapy. Nat. Rev. Cancer 19 (9), 495–509. Available at:. 10.1038/s41568-019-0179-8 31406302 PMC7043073

[B44] Sturm G. Finotello F. Petitprez F. Zhang J. D. Baumbach J. Fridman W. H. (2019). Comprehensive evaluation of transcriptome-based cell-type quantification methods for immuno-oncology. Bioinformatics 35 (14), i436–i445. Available at:. 10.1093/bioinformatics/btz363 31510660 PMC6612828

[B45] van der Leun A. M. Thommen D. S. Schumacher T. N. (2020). CD8+ T cell states in human cancer: insights from single-cell analysis. Nat. Rev. Cancer 20 (4), 218–232. Available at:. 10.1038/s41568-019-0235-4 32024970 PMC7115982

[B46] Wang D. Qian X. Du Y. C. N. Sanchez-Solana B. Chen K. Kanigicherla M. (2023). cProSite: a web based interactive platform for online proteomics, phosphoproteomics, and genomics data analysis. bioRxiv 2023, 543932. Available at:. 10.1101/2023.06.10.543932 PMC11210690 38938288

[B47] Wang X. He Q. Shen H. Xia A. Tian W. Yu W. (2019). TOX promotes the exhaustion of antitumor CD8+ T cells by preventing PD1 degradation in hepatocellular carcinoma. J. Hepatology 71 (4), 731–741. Available at:. 10.1016/j.jhep.2019.05.015 31173813

[B48] Yoshihara K. Shahmoradgoli M. Martínez E. Vegesna R. Kim H. Torres-Garcia W. (2013). Inferring tumour purity and stromal and immune cell admixture from expression data. Nat. Commun. 4, 2612. Available at:. 10.1038/ncomms3612 24113773 PMC3826632

[B49] Yu Z. Du M. Lu L. (2022). A novel 16-genes signature scoring system as prognostic model to evaluate survival risk in patients with glioblastoma. Biomedicines 10 (2), 317. Available at:. 10.3390/biomedicines10020317 35203526 PMC8869708

[B50] Zirlik K. Duyster J. (2018). Anti-angiogenics: current situation and future perspectives. Oncol. Res. Treat. 41 (4), 166–171. Available at:. 10.1159/000488087 29562226

[B51] Zou Z. Tao T. Li H. Zhu X. (2020). mTOR signaling pathway and mTOR inhibitors in cancer: progress and challenges. Cell. and Biosci. 10 (1), 31. Available at:. 10.1186/s13578-020-00396-1 PMC7063815 32175074

