# Supplementary material for: AVEN: a novel oncogenic biomarker with prognostic significance and implications of AVEN-associated immunophenotypes in lung adenocarcinoma
Source: Front Mol Biosci. 2023 Oct 16;10:1265359. doi: 10.3389/fmolb.2023.1265359 (PMC10613694; doi:10.3389/fmolb.2023.1265359)
Supplement: Supplementary file 1 [file DataSheet1.ZIP › supplementary/supplementary data_FMB.docx]

Supplementary Material

**AVEN: A Novel Oncogenic Biomarker with Prognostic Significance and Implications of Aven-associated Immunophenotypes in Lung Adenocarcinoma**

Dengxia Fan^1^, Moses Yang^1^, HyeJung Lee^1^, Jeong Hee Lee^1^, Hong Sook Kim^1*^

*** Correspondence:** Hong Sook Kim: [Hong2kim2@gmail.com](mailto:Hong2kim2@gmail.com)

## Supplementary Figure


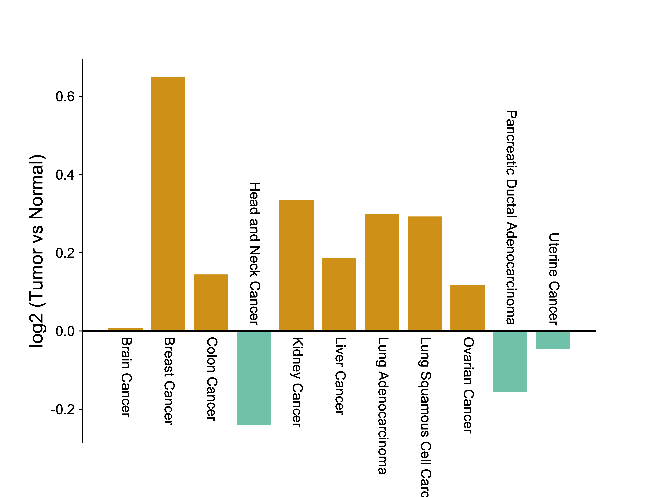


AVEN protein abundance across diverse tumor types


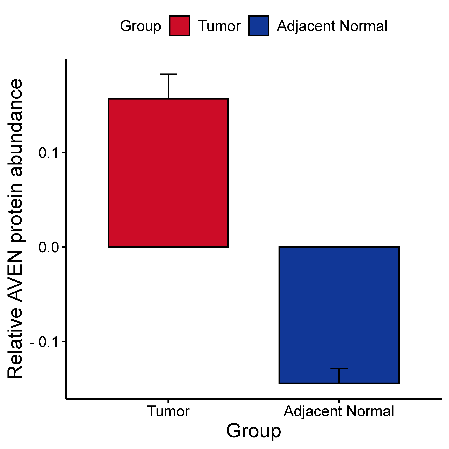

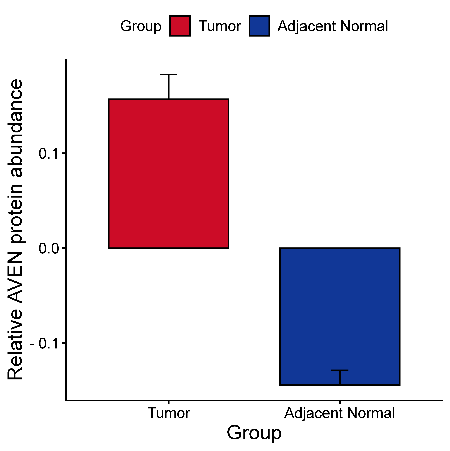


AVEN protein abundance in LUAD

A

B


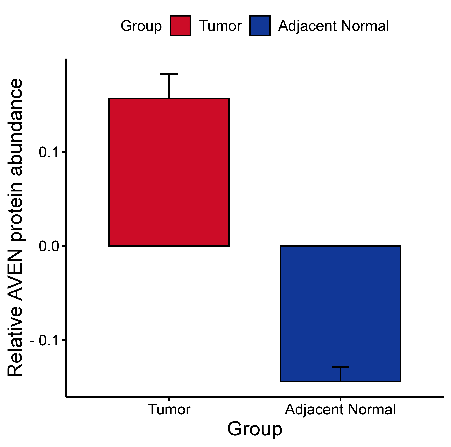


**Supplementary Figure S1. AVEN protein abundance in tumor tissues and normal tissues (A)** AVEN protein abundance across diverse tumor types **(B)** AVEN protein is abundant in tumor tissue compared to adjacent normal tissue in lung adenocarcinoma (LUAD).


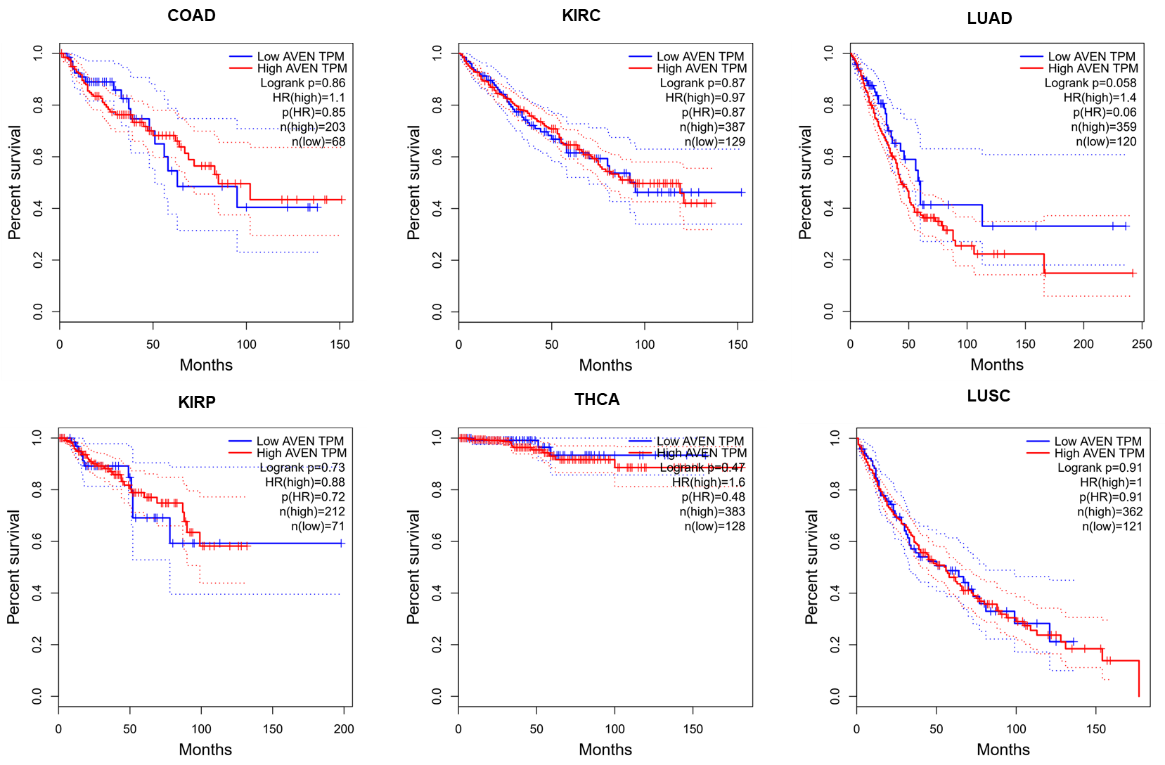


**Supplementary Figure S2.** AVEN-associated **s**urvival analysis using the GEPIA web tool in six different types of cancer, including colon adenoma (COAD), Kidney renal clear cell carcinoma (KIRC), Kidney renal papillary cell carcinoma (KIRP), Lung adenocarcinoma (LUAD), Lung squamous cell carcinoma (LUSC), and Thyroid carcinoma (THCA). The group cutoff value is based on AVEN expression: top 25% and bottom 25%.


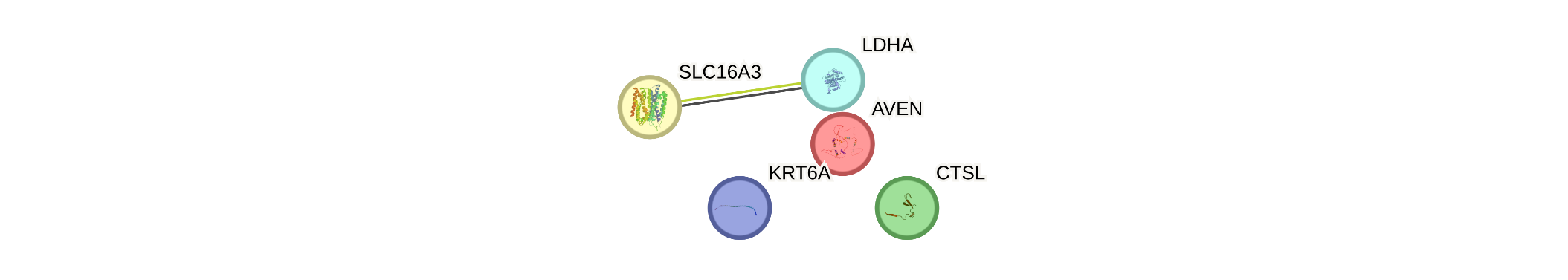


**Supplementary Figure S3.** Protein-protein-interaction (PPI) analysis among AVEN and five genes included in the AVEN-derived prognostic model, namely SLC16A3, KRT6A, LDHA, CDC42EP2, and CTSL. The string web tool (<https://string-db.org/>) was used to explore the proteins interaction among these proteins.
